# Metabolomic Profiling Reveals the Effects of Cu-Ag Nanoparticles on Tomato Bacterial Wilt

**DOI:** 10.3390/metabo15080548

**Published:** 2025-08-13

**Authors:** Weimin Ning, Lei Jiang, Mei Yang, Tianhao Lei, Chan Liu, Fei Zhao, Pan Shu, Yong Liu

**Affiliations:** 1 Agricultural Science College, Xichang University, Xichang 615000, China; ningweimin@hnu.edu.cn (W.N.);; 2 Longping Branch, Biology College, Hunan University, Changsha 410125, China; 3 Key Laboratory of Pest Management of Horticultural Crop of Hunan Province, Hunan Academy of Agricultural Science, Changsha 410125, China

**Keywords:** Cu-Ag nanoparticles, tomato, metabolomics, fatty acyls, organooxygen compounds

## Abstract

**Background:** The bacterial wilt of tomatoes, caused by *Ralstonia solanacearum*, is a soil-borne plant disease that causes substantial agricultural economic losses. Various nanoparticles have been utilized as antibacterial agents to mitigate pathogenic destructiveness and improve crop yields. However, there is a lack of in-depth research on how nanoparticles affect tomato metabolite levels to regulate the bacterial wilt of tomatoes. **Methods**: In this study, healthy and bacterial wilt-infected tomatoes were treated with Cu-Ag nanoparticles, and a metabolomics analysis was carried out. **Results:** The results showed that Cu-Ag nanoparticles had a significant prevention and control effect on the bacterial wilt of tomatoes. Metabolomic analysis revealed that the nanoparticles could significantly up-regulate the expression levels of terpenol lipids, organic acids, and organic oxygen compounds in diseased tomatoes, and enhance key metabolic pathways such as amino acid metabolism, carbohydrate metabolism, secondary metabolite metabolism, and lipid metabolism. These identified metabolites and pathways could regulate plant growth and defense against pathogens. Correlation analysis between the tomato microbiome and metabolites showed that most endophytic microorganisms and rhizospheric bacteria were positively correlated with fatty acyls groups and organic oxygen compounds. **Conclusions:** This study reveals that Cu-Ag nanoparticles can actively regulate the bacterial wilt of tomatoes by up-regulating the levels of lipid metabolism and organic oxygen compounds, providing an important theoretical basis for the application of nanoparticles in agriculture.

## 1. Introduction

Soil-borne plant pathogens can colonize and accumulate in the soil through synergistic symbiotic relationships, posing a serious threat to the root and stem tissues of plants and causing enormous agricultural economic losses [1,2,3]. *Ralstonia solanacearum* (*R. solanacearum*) is the second most dangerous phytopathogen worldwide among soil-borne plant pathogens, and is a Gram-negative bacterium, which causes bacterial wilt [4]. In tomatoes, under the impact of heavy infection, the *R. solanacearum* population can accumulate to 10^3^–10^6^ in soil and 10^8^ cfu/g of plant tissue, which leads to a yield loss of 90% [5]. *R. solanacearum* residing in soil and contaminated water can penetrate plant roots via wounds or natural openings; the pathogen subsequently colonizes the intercellular spaces of the root and infiltrates the xylem vessels, subsequently disseminating upward into the stems through the xylem [6,7,8]. The abundance of pathogens and exopolysaccharide slime generated by pathogenic bacteria resulted in diminished sap flow in xylem vessels, thereby inducing wilting symptoms in tomatoes [9,10]. Nanotechnology is one of the fastest-developing sciences of the 21st century, which can be utilized in soil, seeds, roots, and foliage to provide protection for plants against pathogens such as *R. solanacearum*. Numerous nanoparticles, including Ag nanoparticles [11,12], Zn nanoparticles [13,14], chitosan nanoparticles [15], MgO nanoparticles [16], silica nanoparticles [17,18,19], Cu nanoparticles [20,21], and Mn nanoparticles [22], have been extensively studied and demonstrated remarkable antibacterial properties against *R. solanacearum*.

Metabolomics could provide a comprehensive analysis of the metabolite composition of plants, which is extensively employed to explore the molecular mechanisms of plants exposed to various environmental conditions [23,24]. Recently, metabolomics analyses have been frequently employed to investigate the molecular responses of plants to various types of nanoparticles. Tian et al. observed a considerable change in the metabolite profile of pakchoi following foliar application of SiO_2_-NPs. Significant increases in the relative abundance of sugars and sugar alcohols, fatty acids, and small organic acids have been identified [25]. Stałanowska et al. observed that ZnO NPs enhanced the amount of metabolites in pea seedlings that participate in the TCA cycle and the aspartate–glutamate pathway. ZnO NPs lowered the amount of malate and total amino acids in wheat [26]. Additionally, research also demonstrate that nanomaterials could regulate the metabolism of starch and sucrose, secondary metabolites, nitrogen, phenolic acids and aldehydes, sulfur, propanoate, amino acids, and fatty acids in plants, most of which could influence crop phenotypes [27,28,29,30]. More importantly, some metabolites significantly influence crop growth, development, and various physiological activities. They play a crucial role in regulating defense biological processes and regulating microbial communities in response to abiotic and biotic stresses, thereby providing positive effects to plants [31,32,33].

Plant roots can be categorized into two distinct microbial compartments based on the surrounding soil conditions: the rhizosphere and the endosphere [34]. Bacteria compose the dominant microbial community in plants, which accounts for the majority constitution of the two microbial compartments observed in crops [35]. Endophytic bacteria that live inside plant tissues have the capacity for continuous contact with plant cells and metabolites, therefore they can generate direct beneficial effects for the plant [36,37]. The majority of endophytic microbes come from the rhizosphere soil and plant surface. Rhizosphere bacteria inhabit the region surrounding plant roots, which is a microhabitat established through the interaction between plant root exudates and soil microorganisms [38,39]. The composition of the plant microbiome is essential for maintaining the best plant health, and it also plays an essential function in providing resistance against various pathogens, including fungi, bacteria, and viruses [40,41].

The interactions between metabolites, microorganisms, and plants, including their roots, stems, leaves, and fruits, are complicated and diverse. The relationship between them has a significant impact on the high yield of plants and plays a crucial role in disease resistance [42,43]. The integration of metabolomics with microorganisms can enhance the comprehension of the interactions between plant microbiome metabolites and hosts toward various conditions [44]. Specifically, it can offer a comprehensive, extensive and complicated understanding of the mechanisms by which plants respond to nanoparticles [45]. Furthermore, some soil microorganisms have the ability to promote plant growth through the production of hormones [46]. There are some functional microorganisms that can enhance the synthesis of secondary metabolites, including antioxidants and antimicrobials, thereby inhibiting the growth and spread of pathogens to protect plant health [47,48]. The current study regarding the interactions between metabolism and the endophytic and rhizosphere microbial communities of plants after exposure to nanoparticles remains limited. There is a need to evaluate the alterations between plant metabolism, microorganisms, pathogens, and nanoparticles.

The objective of this study is to perform a more thorough investigation into the impact of nanoparticles on the metabolites of plants. The laboratory-synthesized Cu-Ag nanoparticles were evaluated on healthy tomato plants and tomatoes infected by *R. solanacearum*. In our laboratory’s previous study, Cu-Ag nanoparticles exhibited an average size of 79.24 nm, a polydispersity index of 0.349, and exceptional dispersion; notably, Cu-Ag nanoparticles significantly protected tomatoes against bacterial wilt disease caused by *R. solanacearum* in both curative and protective pot trials conducted at 10 and 20 days in a greenhouse environment [49]. Liquid chromatography-tandem mass spectrometry was utilized to explore the alterations of metabolomes in healthy and infected tomatoes treated with nanoparticles. The potential relationship between bacterial community composition and metabolites was also investigated. This will provide a comprehensive understanding of the physiological interactions among plant pathogens, microorganisms, metabolites, and nanoparticles.

## 2. Materials and Methods

### 2.1. Materials

The tomato seeds (cv. zuanhongmeina) and *Ralstonia solanacearum* (*RS556*) were stored in the Key Laboratory of Pest Management of Horticultural Crop of Hunan Province, Hunan Academy of Agricultural Science; Cu-Ag nanoparticles were synthesized based on our previous study [44]. Thiodiazole-copper was purchased from Zhejiang Longwan Chemicals Co., Ltd., Zhejiang, China; methanol (HPLC grade, purity > 99.9%), acetonitrile (HPLC grade, purity > 99.95%), and formic acid (HPLC grade, purity > 99%) were purchased from Fisher Chemical Co., Ltd., Middleton, WI, USA; cholic acid-D4 (HPLC grade, purity > 98%) and D-luciferin (BR grade, purity > 99%) were purchased from Shanghai yuanye Bio-Technology Co., Ltd., Shanghai, China; succinic acid-d4 (HPLC grade, purity > 98%) was purchased from Sigma-Aldrich Trading Co., Ltd., St. Louis, MO, USA; and purified water was purchased from Hangzhou Wahaha Group Co., Ltd., Hangzhou, China.

### 2.2. Plant Culture and Treatment

The experimental design was based on our previous research results [50,51]. Tomato seeds were planted in plastic pots containing nutritious soil (Jiangsu Jiafeng Agricultural Development Co., Ltd., Jiangsu, China). After seven days, uniformly growing seedlings were transplanted into individual pots. Plants were grown under controlled conditions: 16 h light/8 h dark cycles, 27 ± 2 °C day/night temperature, and 60% relative humidity. Following 30 days of growth, uniformly sized tomato seedlings were randomly assigned to two groups, healthy and infected, and each group was split into three more groups (subgroups). The infected groups include the control diseased (infected with *R. solanacearum*) (D6), diseased treated with Cu-Ag nanoparticles (D7), and diseased treated with thiodiazole-copper (D8). The healthy groups included control healthy (treated with sterile water) (D9), healthy treated with Cu-Ag nanoparticles (D10), and healthy treated with thiodiazole-copper (D11). The groups D6, D7, and D8 received 15 mL of freshly prepared *R. solanacearum* suspension (OD_600_ = 0.1) applied to the roots, while healthy control groups D9, D10, and D11 received 15 mL of sterile water. After 48 h, treatments were applied: groups D7 and D10 received 20 mL of Cu-Ag nanoparticles (diluted to 20 μL/mL based on previous research), groups D8 and D11 received 20 mL of thiodiazole-copper (diluted 500-fold per manufacturer instructions), and control groups D6 and D9 received the same volume (20 mL) of sterile water. The treated plants were grown before being harvested for further analysis.

After treatment, they were grown for 15 days, and only then were the roots collected. The tomato roots were carefully extracted from the pot and gently shaken to eliminate as much of the bulk soil as possible. The roots were transferred to a sterile PBS buffer (pH 7.0). After careful shaking, the roots were moved to a new PBS buffer. The shaking steps were performed twice, for 20 min each time. The root samples were quickly collected and immediately frozen in liquid nitrogen and subsequently stored at −80 °C.

### 2.3. Metabolite Extraction

A 30 mg sample of tomato root was accurately weighed and placed into a 2 mL Eppendorf microcentrifuge tube, which contained 300 μL of prechilled methanol–water (V:V = 7:3, containing mixed internal standard (a mixture including 4 μg/mL L-valine-d8, 4 μg/mL succinic acid-d4, 4 μg/mL 2-chloro-L-phenylalanine, 4 μg/mL D-luciferin, and 4 μg/mL cholic acid-D4). After incubation at −40 °C for 2 min, the freeze-dried roots were thoroughly ground into a fine powder. The samples were subsequently sonicated in an ice-water bath for 30 min, and the mixtures were stored at −40 °C for 2 h. Finally, the mixtures were centrifuged for 20 min at 3000 rpm (TGL-16MS, Shanghai Lu Xiangyi Centrifuge Instrument Co., Ltd., Shanghai, China) and 4 °C. QC samples were prepared by mixing aliquots of all of the samples to create a pooled sample. The final supernatant was transferred to the LC vial for liquid chromatography-tandem mass spectrometry (LC-MS/MS)) analysis using the Waters ACQUITY UPLC I-Class plus/Thermo QE HF system (Thermo Fisher Scientific Co., Ltd., Middleton, WI, USA). Briefly, the chromatographic separation was achieved by an ACQUITY UPLC HSS T3 column (100 mm × 2.1 mm, 1.8 um). The binary gradient elution system consisted of (A) water (containing 0.1% formic acid, *v/v*) and (B) acetonitrile (containing 0.1% formic acid, *v/v*) and separation was achieved using the following gradient: 0–2 min, 5% B; 4–8 min, 30–50% B; 8–10 min, 50–80% B; 10–14 min, 80–100% B; 14–15 min, 100% B; and 15.1–16 min, 5% B. All of the samples were kept at 10 °C during the analysis. The column temperature was 45 °C, the injection volume was 3 μL, and the flow rate was 0.35 mL/min [52,53,54]. Five biological replicates for each tomato root treatment were analyzed. The mass range was from *m*/*z* 100 to 1000. The resolution was set at 70,000 for the full MS scans and 17,500 for HCD MS/MS scans. The collision energy was set at 10, 20, and 40 eV. The mass spectrometer operated as follows: spray voltage, 3800 V (+) and 3200 V (−); sheath gas flow rate, 35 arbitrary units; auxiliary gas flow rate, 8 arbitrary units; capillary temperature, 320 °C; Aux gas heater temperature, 350 °C; S-lens RF level, 50.

### 2.4. Metabolomic Analysis

The mass spectrum raw data were analyzed employing the Progenesis QI v3.0 program (Nonlinear, Dynamics, Newcastle, UK) for baseline filtering, peak identification, integral, retention time correction, peak alignment, and QI normalization. Main parameters of 5 ppm precursor tolerance, 10 ppm product tolerance, and 5% product ion threshold were applied. The investigation of the metabolites from the tomato root samples was performed qualitatively and quantitatively by using the self-constructed metabolome database (OE biotech Co., Ltd., Shanghai, China). The leveling of metabolites occurred relative to the internal control. The unsupervised principal component analysis (PCA), partial least squares discriminant analysis (PLS-DA), and orthogonal partial least squares discriminant analysis (OPLS-DA) were performed to find differential metabolites [55,56]. After evaluating the root metabolites through fold-change scores, the amounts of up-regulated and down-regulated metabolites in different treatments were illustrated based on the thresholds set at FC ≥ 2.0 and *p* < 0.05 [57]. The unsupervised hierarchical clustering analysis (HCA) of differential metabolites from the fold-change heat map was processed [58]. The differentially expressed metabolites (DEMs) were assigned Kyoto Encyclopedia of Genes and Genomes (KEGG) function annotations for enrichment analysis [59]. The biological pathway of tomato root metabolites was investigated utilizing the KEGG pathway and KEGG orthology [60]. The analysis of the interactions between metabolism and microorganisms employs mixOmics and NetworkX [61]. A one-way ANOVA followed by a Fisher’s least significant difference method (Fisher’s LSD, *p* value set as 0.05) was conducted to identify the significantly altered metabolites.

## 3. Results

### 3.1. Effect of Nanoparticles on the Overall Metabolic Profile of Tomato Root

**Figure 1:**
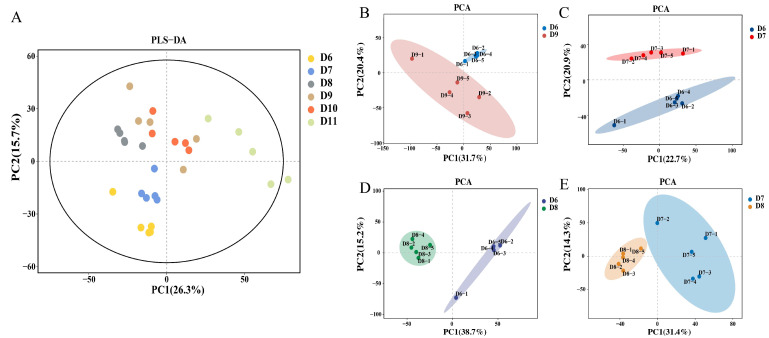
Scores plot of partial least squares discriminant analysis (PLS-DA) of metabolites in all tomato samples (n = 5) (**A**). Principal component analysis (PCA) of root metabolites. D6 vs. D9 (**B**). D6 vs. D7 (**C**). D6 vs. D8 (**D**). D7 vs. D8 (**E**). Abbreviations: control infected tomatoes (D6); infected tomato roots treated with Cu-Ag nanoparticles (D7); infected tomato roots treated with thiodiazole-copper (D8); control healthy tomatoes (D9); healthy tomatoes treated with Cu-Ag nanoparticles (D10); and healthy tomatoes treated with thiodiazole-copper (D11).

In our previous work, the Cu-Ag nanoparticles displayed a diminutive size and superior dispersion, demonstrating the exceedingly effective protection of tomatoes against *R. solanacearum* [44]. To assess the impact of nanoparticles on plant metabolism, the metabolomes of both healthy and diseased tomatoes treated with nanoparticles were analyzed. To illustrate the general distinctions among various treated tomato plants, the metabolites from different tomato samples were standardized and analyzed using partial least squares discriminant analysis (PLS-DA). The PLS-DA score demonstrated a clear separation among tomato root samples that responded to various treatments, including both healthy and infected tomatoes (Figure 1A–E. To further analyze the variation present in the tomato root and identify differential metabolites, the orthogonal partial least squares discriminant analysis (OPLS-DA) was performed. The OPLS-DA score showed a clear separation among tomato root samples, indicating that Cu-Ag nanoparticles altered the metabolic profiles of the infected tomato roots (Figures S1 and S2). Overall, the differences among tomato root samples indicated that the application of Cu-Ag nanoparticles affected the metabolic structure of roots.

### 3.2. Number of Metabolite Changes Induced by Cu-Ag Nanoparticles

To enhance understanding of the influence of nanoparticles on the metabolomics of tomatoes, a Venn analysis was performed on differential metabolites. The analysis identified 21 compounds that were overlapped throughout D6 (control diseased tomatoes) vs. D7 (diseased tomatoes treated with Cu-Ag nanoparticles), D6 vs. D8 (diseased tomatoes treated with thiodiazole-copper), and D7 vs. D8. More unique metabolites were identified in D6 vs. D8 (337) compared with D6 vs. D7 (50), and there were 127 metabolites shared in these two infected tomato root comparison groups (Figure S3A). In addition, the root contained only 56 unique compounds in D9 (control healthy tomatoes) vs. D10 (healthy tomatoes treated with Cu-Ag nanoparticles), whereas a higher amount of distinctive metabolites were identified in D9 vs. D11 (healthy tomatoes treated with thiodiazole-copper) (254) (Figure S3B). The results indicated that varying levels of metabolites were significantly modified in different comparison groups of tomato roots (Figure S3C). The analysis of the differential metabolites in tomatoes showed the presence of 53 down-regulated and 148 up-regulated between D7 and D6; 447 down-regulated and 327 up-regulated between D8 and D6; 80 down-regulated and 60 up-regulated between D10 and D9; and 258 down-regulated and 67 up-regulated between D10 and D7 (Figure S4).

### 3.3. Analysis of Differential Root Metabolites Between Nanoparticle-Treated and Thiodiazole-Copper-Treated Plants

**Figure 2:**
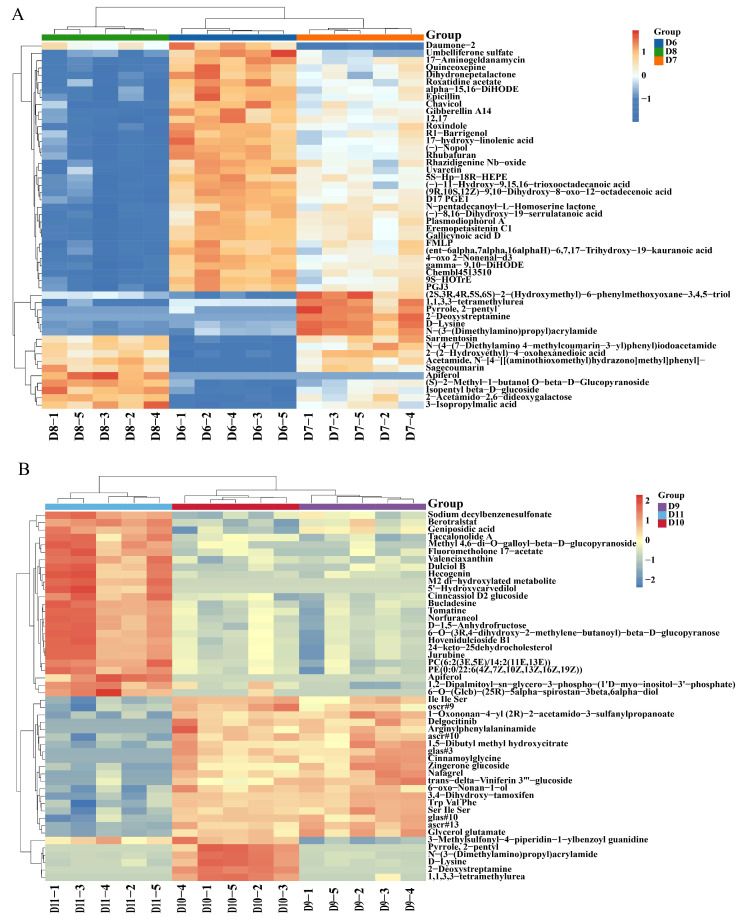
Hierarchical cluster analysis of the top 50 differentially expressed metabolites in infected tomato roots (**A**) and healthy tomato roots (**B**). Each column represents a root sample, and each row indicates a differentially expressed metabolite. The color gradient transitioning from blue to red represents the varying abundance of differentially expressed metabolites, from low to high levels.

The hierarchical cluster analysis (HCA) demonstrated the composition and alterations of metabolites in the infected tomato roots subjected to Cu-Ag nanoparticles and thiodiazole-copper. The main metabolites of diseased tomatoes exhibiting notable alterations comprised fourteen fatty acyls, six organooxygen compounds, seven prenol lipids, and five carboxylic acids (Figure 2A, Table S1). The alterations in metabolites for healthy tomato roots displayed variation. The Cu-Ag nanoparticles also modified several metabolites in the roots of healthy tomatoes. These metabolites included ten carboxylic acids, six steroids and steroid derivatives, five prenol lipids, and five organooxygen compounds (Figure 2B, Table S2). The HCA of all samples indicated specific variations in the metabolite clustering of healthy and infected tomato roots treated with Cu-Ag nanoparticles.

**Figure 3:**
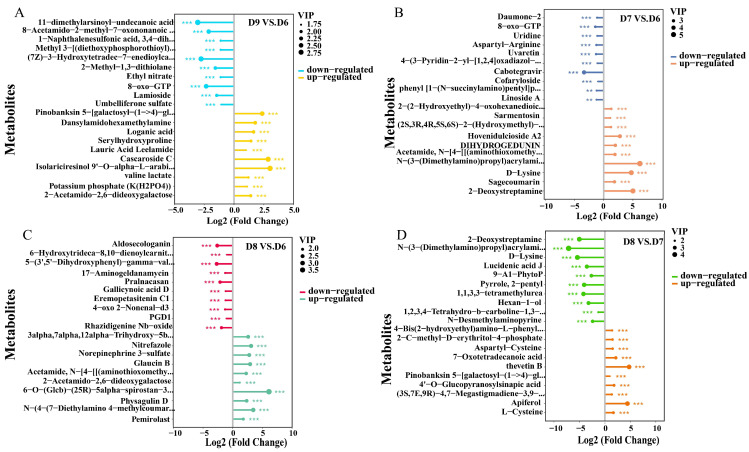
Analysis of the up-regulated and down-regulated metabolites in tomato roots using Lollipop Map. D9 vs. D6 (**A**). D7 vs. D6 (**B**). D8 vs. D6 (**C**). D8 vs. D7 (**D**). ** *p* ≤ 0.01 and *** *p* ≤ 0.001.

We conducted a comparative analysis of the differentially expressed metabolites in various comparison groups using Lollipop Map. The significant down-regulation was observed in the metabolism of fatty acids (11-dimethylarsinoyl-undecanoic acid, 8-acetamido-2-methyl-7-oxononanoic acid, and (7Z)-3-hydroxytetradec-7-enedioylcarnitine), and organic acids (serylhydroxyproline and valine lactate) and prenol lipids (loganic acid and lauric acid leelamide) showed up-regulation in D9 vs. D6 (Figure 3A). The metabolism of prenol lipids (hovenidulcioside A2 and DIHYDROGEDUNIN), organic Acids (N-(3-(dimethylamino) propyl) acrylamide and D-Lysine), organooxygen compounds ((2S,3R,4R,5S,6S)-2-(hydroxymethyl)-6-phenylmethoxyoxane-3,4,5-triol and 2-deoxystreptamine) were up-regulated in D7 vs. D6 (Figure 3B).

Moreover, fatty acids (6-hydroxytrideca-8,10-dienoylcarnitine and gallicynoic acid D) displayed significant down-regulation, and steroids (3alpha,7alpha,12alpha-Trihydroxy-5beta-cholestan-24-one, 6-O-(Glcb)-(25R)-5alpha-spirostan-3beta,6alpha-diol, and physagulin D) were up-regulated in D8 vs. D6 (Figure 3C). Flavonoids (pinobanksin 5-[galactosyl-(1->4)-glucoside] and apiferol) were up-regulated in D8 vs. D7 (Figure 3D). The metabolite variations in reaction to Cu-Ag nanoparticle treatments for healthy tomato roots were different. Carboxylic acids metabolites (ectoine, D-lysine, and N-(3-(dimethylamino)propyl)acrylamide) were up-regulated in D10 vs. D9. Organooxygen compounds (zingerone glucoside, validamycin A and ascr#13) and carboxylic acids (arginylphenylalaninamide, cinnamoylglycine, and 1-oxononan-4-yl (2R)-2-acetamido-3-sulfanylpropanoate) were down-regulated in D11 vs. D9 (Figure S5). The results indicate that the metabolites associated with prenol lipids, carboxylic acids, and organooxygen compounds exhibited major alterations after Cu-Ag nanoparticle treatments.

### 3.4. KEGG Pathway Enrichment Analysis in Tomato Roots

**Figure 4:**
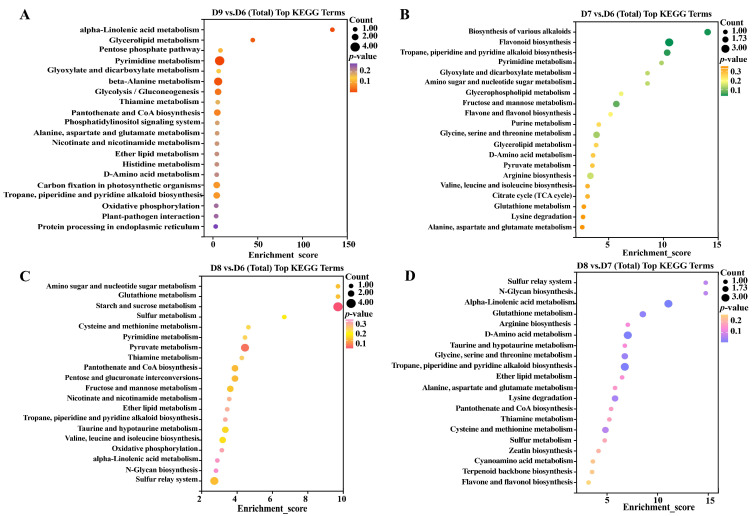
KEGG enrichment analysis of the pathways in infected tomato roots. The X-axis represented the route name, while the Y-axis denoted the enrichment score. The bubble’s size reflects the number of involved DEMs. D9 vs. D6 (**A**). D7 vs. D6 (**B**). D8 vs. D6 (**C**). D8 vs. D7 (**D**).

The Kyoto Encyclopedia of Genes and Genomes (KEGG) pathway enrichment analysis was utilized to determine the top 20 pathways in different groups. The lipid metabolism (glycerolipid metabolism and ether lipid metabolism) was up-regulated; meanwhile, amino acid metabolism pathways (alanine, aspartate and glutamate metabolism, D-amino acid metabolism, and beta-alanine metabolism), secondary metabolites (tropane, piperidine, and pyridine alkaloid biosynthesis), and environmental adaptation (plant–pathogen interactions) were significantly down-regulated in D9 vs. D6 (Figure 4A, Table S3).

In comparison to the blank infected tomato, the Cu-Ag nanoparticle treatment group displayed an up-regulation of six amino acid metabolism pathways (such as lysine degradation and arginine biosynthesis), five carbohydrate metabolism pathways (such as pyruvate metabolism and the citrate cycle), and two lipid metabolism pathways, while the biosynthesis of various alkaloids, flavone and flavonol biosynthesis, and pyrimidine metabolism were down-regulated (Figure 4B, Table S4). The primary pathways of diseased tomatoes that exhibited significant up-regulation after thiodiazole-copper treatment included the metabolism of four amino acids and three carbohydrates. (Figure 4C, Table S5). The primary alteration of pathways was amino acid metabolism in D8 vs. D7 (Figure 4D, Table S6).

The top pathways exhibiting the highest enrichment among the healthy tomato roots were analyzed. There were six pathways of amino acid metabolism up-regulated in D10 vs. D9. A noteworthy down-regulation was observed in the amino acid metabolism pathway in D11 vs. D9. Only the pyrimidine metabolism and alpha-linolenic acid metabolism pathways were up-regulated, while all other pathways were down-regulated in D11 vs. D10 (Figure S5, Tables S7–S9). Consequently, the utilization of Cu-Ag nanoparticles primarily focuses on the regulation of amino acid and carbohydrate metabolism.

### 3.5. Correlation Analysis Between Metabolites and Microorganisms in Tomato

**Figure 5:**
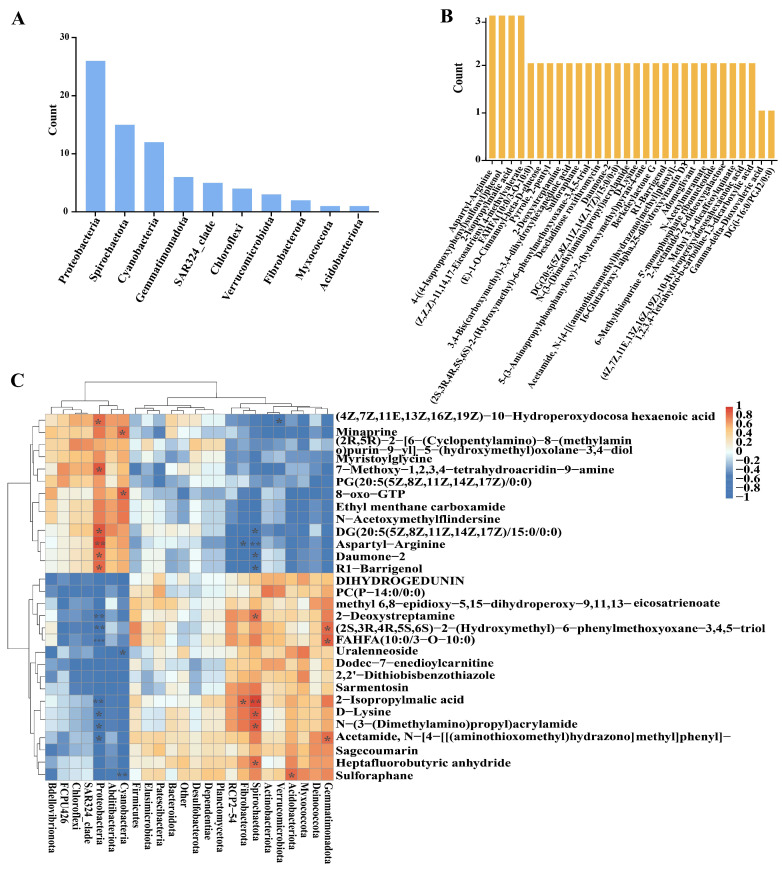
Analysis of endophytic microbiota (**A**) and metabolomic (**B**) characteristics in D7 vs. D6. The notable connections were determined based on the *p* value, and the frequency of each disparity in each group was individually assessed. The count reflects the frequency of associated items, arranged in decreasing order. Hierarchical cluster analysis of the correlation between metabolites and endophytic microbial communities (**C**). The horizontal axis shows bacterial communities at the phylum level, while the vertical axis illustrates the metabolic products. * *p* ≤ 0.05; ** *p* ≤ 0.01; and *** *p* ≤ 0.001.

The correlation analysis was performed based on Spearman’s algorithm to acquire a comprehensive understanding of the relationship between metabolites and microbes. In the comparison between control infected tomatoes and infected tomatoes treated with Cu-Ag nanoparticles, a total of 10 endophytic bacterial phyla were identified, exhibiting significant correlations with 30 root metabolites (Figure 5A,B). The correlations were illustrated via a heatmap. *Proteobacteria* exhibits a negative correlation with the metabolites fatty acyls (FAHFA (10:0/3-O-10:0) and 2-isopropylmalic acid) and organooxygen compounds (2-deoxystreptamine, (2S,3R,4R,5S,6S)-2-(hydroxymethyl)-6-phenylmethoxyoxane-3,4,5-triol, and daumone−2), which were up-regulated by the application of nanoparticles. There is a positive correlation between *Gemmatimonadota and Spirochaetota* with the three organooxygen compounds. The application of nanoparticles increased the relative abundance of *Gemmatimonadota, Fibrobacterota*, and *Spirochaetota*, which demonstrates a significant positive correlation with FAHFA (10:0/3-O-10:0) and 2-isopropylmalic acid, respectively. *Spirochaetota* demonstrated a notable positive correlation with organic acids (D-lysine and N-(3-(dimethylamino) propyl) acrylamide) (Figure 5C).

**Figure 6:**
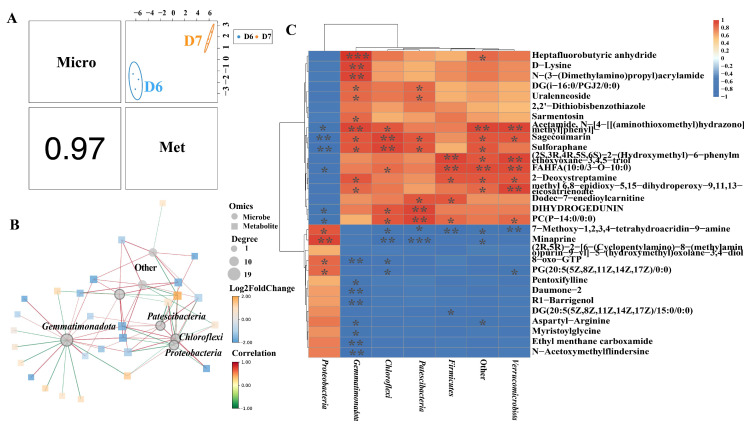
Comprehensive analysis of the rhizosphere microbiome at the phylum level and metabolomic profiles in D7 vs. D6. Correlation plot showing the association among components (**A**). The top right graph illustrates the distribution of metabolite samples. The bottom left graphic illustrates the correlation coefficient of the sample distribution. Analysis of the network relationship (**B**). The highest correlations between metabolomics and the microbiota were identified. Squares represent metabolites, while circles indicate microbes. The color of the line reflects the correlation coefficient. Hierarchical cluster analysis of the connection between metabolites and microorganisms (**C**). The horizontal axis represents the microorganisms, and the vertical axis shows metabolites. Red denotes a positive connection, while blue signifies a negative correlation. The intensity of color reflects the degree of association. * *p* ≤ 0.05; ** *p* ≤ 0.01; and *** *p* ≤ 0.001.

A correlation analysis was conducted at the bacterial phylum level of the rhizosphere. The correlation value between the metabolites and bacteria is 0.97 (Figure 6A). The microbiota-metabolite network revealed that *Proteobacteria, Gemmatimonadota, Chloroflexi,* and *Patescibacteria* were the key taxa significantly correlated with root metabolites (Figure 6B). *Proteobacteria* exhibited a positive correlation with organoheterocyclic compounds (7-methoxy-1,2,3,4-tetrahydroacridin-9-amine and minaprine), and *Verrucomicrobiota, Patescibacteria, Chloroflexi*, and *Firmicutes* demonstrated a negative relationship with them. Proteobacteria showed a negative association with fatty acyls (FAHFA (10:0/3-O-10:0)) and lipids (DIHYDROGEDUNIN and PC (P-14:0/0:0)). The abundance of three metabolites in the diseased tomatoes increased after nanoparticle treatments. *Gemmatimonadota* demonstrated a positive correlation with fatty acyls (DG(i-16:0/PGJ2/0:0)) and organic acids (D-lysine and N-(3-(dimethylamino)propyl)acrylamide). *Verrucomicrobiota* and *Firmicutes* had a positive correlation with organooxygen compounds ((2S,3R,4R,5S,6S)-2-(hydroxymethyl)-6-phenylm ethoxyoxane-3,4,5-triol and 2-deoxystreptamine) (Figure 6C). As a result, metabolic activities in the infected tomato roots were associated with microbial communities under the impact of nanoparticles.

## 4. Discussion

Tomatoes (*Solanum lycopersicum*) are one of the most economically valuable crops worldwide, but they are highly susceptible to the severe threat of bacterial wilt [62]. An increasing number of nanomaterials are being developed and optimized for the efficient control of plant diseases, owing to their unique physical and chemical properties [1,63]. Current studies on the alteration of plant metabolism following nanoparticle exposure are still limited. Metabolomic studies were performed to investigate the influence of nanomaterials on both healthy tomato plants and those infected with *Ralstonia solanacearum.* Data showed that 53 metabolites were down-regulated and 148 were up-regulated in infected tomatoes treated with nanoparticles, whereas 60 metabolites exhibited up-regulation and 80 displayed down-regulation in healthy tomatoes treated with nanoparticles. The application of nanoparticles induces alterations in tomato metabolites.

*R. solanacearum* invasion induced an up-regulation of fatty acids and a down-regulation of organic acids and prenol lipids. Organic acids, as bioactive molecules analogous to antibiotics, exhibit potent antibacterial and antifungal activities [64,65]. Song et al. found that organic acids enhances plant biomass, increase chlorophyll content, and stimulate root development in Hydrangea macrophylla [66]. Prenol lipids exhibit a variety of biological actions, such as enhancing direct plant defense systems and attracting beneficial pollinators [67]. Treatment with Cu-Ag nanoparticles promoted the up-regulation of prenol lipids and organic acids in the infected tomato. Organooxygen compounds, including carbohydrates, have shown antioxidant and antibacterial effects [68]. An increase in organooxygen compounds has been discovered in infected tomatoes following the application of nanoparticles. These data indicate that Cu-Ag nanoparticles may enhance certain metabolites, hence providing protection to plants against pathogens.

The plant’s inherent defense mechanisms can regulate its metabolic response to protect itself against external stresses [69,70]. The analysis of pathway enrichment showed that the invasion of *R. solanacearum* up-regulated pathways related to environmental adaptability, amino acid metabolism, nucleotide metabolism, and signal transduction, whereas lipid metabolism was down-regulated. The nanoparticles significantly promoted the up-regulation of lipid metabolism pathways. Lipids perform multiple functions in plant cells, including signal transmission, membrane composition, energy storage, and the provision of metabolic resources [71,72]. Le et al. found that chlorogenic acid can achieve its antibacterial effects by enhancing lipid metabolism [73]. Our study revealed that six amino acid metabolism pathways and five carbohydrate metabolism pathways were up-regulated in infected tomato plants after treatment with nanoparticles. Amino acid metabolism is intricately connected to stress signaling and defense responses in the plant systemic immune response [74,75]. Carbohydrate metabolism may affect bacterial chemotaxis and eliminate infections; Du et al. noted that trans-cinnamaldehyde has antibacterial properties against Escherichia coli via enhancing carbohydrate and energy metabolism [76]. Our findings indicate that Cu-Ag nanoparticles promoted the up-regulation of lipid, amino acid, and glucose metabolism pathways in infected tomatoes, which may be crucial to maintaining balance between plant development and pathogen burden.

We analyzed the correlation between metabolites and endophytic microorganisms in infected tomatoes treated with Cu-Ag nanoparticles. *Proteobacteria* showed a negative correlation with fatty acyls, while a positive correlation was discovered with *Gemmatimonadota, Fibrobacterota,* and *Spirochaetota*. Fatty acids are organic compounds containing carboxylic acid groups; plants are capable of synthesizing fatty acids as a defensive strategy against diseases, particularly multidrug-resistant bacteria [77]. Furthermore, fatty acids possess synergistic antibacterial properties against both Gram-positive and Gram-negative bacteria when combined with penicillin and aminoglycosides [78]. In our study, fatty acid levels increased in infected tomato roots after exposure to Cu-Ag nanoparticles. The results indicate the nanoparticles may affect the relative abundance of rhizosphere bacteria, thereby influencing fatty acid metabolism to protect tomatoes.

The metabolites of fatty acyls (DG(i-16:0/PGJ2/0:0) and FAHFA (10:0/3-O-10:0)) and prenol lipids (DIHYDROGEDUNIN) were up-regulated in infected tomatoes after the application of nanoparticles. The rhizosphere bacteria, including *Verrucomicrobiota, Patescibacteria, Chloroflexi, Gemmatimonadota,* and *Firmicutes*, have a positive correlation with fatty acyls. Verrucomicrobiota and Firmicutes have a positive relation to organooxygen compounds. A notable positive correlation exists between the endophyte microorganisms Gemmatimonadota and Spirochaetota and the two organooxygen compounds ((2S,3R,4R,5S,6S)-2-(hydroxymethyl)-6-phenylmethoxyoxane-3,4,5-triol and 2-deoxystreptamine), as well as another organooxygen compound (daumone-2). The majority of endophytic and rhizosphere bacteria have a positive association with fatty acyls and organooxygen compounds. Organooxygen compounds and fatty acids have shown the potential to impact antibacterial efficacy [68,77,78]. The interaction between metabolites and bacteria after the application of nanoparticles in diseased tomatoes may provide insights into the regulatory mechanisms of nanomaterials that influence tomato diseases. Subsequent research may concentrate on examining these associations and identifying a category of key regulatory metabolites.

## 5. Conclusions

This study systematically investigated the impact of Cu-Ag nanoparticles on the metabolic profiles of tomato plants infected with *R. solanacearum*. The results revealed that these nanoparticles significantly modulated the metabolic landscape of tomato roots, evidenced by the up-regulation of prenol lipids, organic acids, and organooxygen compounds. Moreover, Cu-Ag nanoparticles were found to promote the regulation of key biological pathways, including amino acid metabolism, carbohydrate metabolism, secondary metabolism, and lipid metabolism, in the roots of infected tomatoes. Notably, a significant positive correlation was observed between the majority of endophytic microbiota and rhizospheric bacterial communities with fatty acyls and organooxygen metabolites. These findings not only deepen the understanding of how nanoparticles influence plant metabolome reprogramming but also provide novel mechanistic insights into the application of nanomaterials for combating plant bacterial pathogens.

## Data Availability

The raw reads have been deposited in NCBI. The metabolomics data reported in this paper have been deposited in the OMIX, China National Center for Bioinformation/Beijing Institute of Genomics, Chinese Academy of Sciences (https://ngdc.cncb.ac.cn/omix, accession No. OMIX007247; accessed on 1 January 2025).

## Supplementary Materials

The following supporting information can be downloaded at https://www.mdpi.com/article/10.3390/metabo15080548/s1: Figure S1: Score plot of orthogonal partial least squares discriminant analysis (OPLS-DA) of tomato root metabolites. D9 vs. D6 (A). D7 vs. D6 (B). D8 vs. D6 (C). D7 vs. D8 (D). Figure S2: Score plot of orthogonal partial least squares discriminant analysis (OPLS-DA) of tomato root metabolites. D10 vs. D9 (A). D11 vs. D9 (B). D11 vs. D10. Figure S3: Venn diagram displaying unique and shared metabolites (DAMs) in diseased tomato roots (A) and healthy tomato roots (B). Upset diagram of DAMs in infected tomato roots and healthy roots (C). Figure S4: Analysis of differentially accumulated metabolites (DAMs) displaying up-regulation and down-regulation in sick tomato roots (A), healthy tomato roots (B), and the comparative groups of healthy and infected roots (C). Figure S5: Analysis of the up-regulated and down-regulated metabolites in tomato roots using Lollipop Map. D9 vs. D10 (A). D9 vs. D11 (B). D10 vs. D11 (C). Figure S6: KEGG enrichment analysis of pathways in healthy tomato roots. The X-axis represents the -log10 p value of each pathway, and the Y-axis denotes the names of the various pathways. D9 vs. D10 (A). D9 vs. D11 (B). D10 vs. D11 (C). Table S1: Metabolites along with their respective classification levels in diseased tomatoes. Table S2: Metabolites along with their respective classification levels in healthy tomatoes. Table S3: Regulation of metabolites and their corresponding classification levels in the comparison group D9 vs. D6. Table S4: Regulation of metabolites and their corresponding classification levels in the comparison group D7 vs. D6. Table S5: Regulation of metabolites and their corresponding classification levels in the comparison group D8 vs. D6. Table S6: Regulation of metabolites and their corresponding classification levels in the comparison group D8 vs. D7. Table S7: Regulation of metabolites and their corresponding classification levels in the comparison group D10 vs. D9. Table S8: Regulation of metabolites and their corresponding classification levels in the comparison group D11 vs. D9. Table S9: Regulation of metabolites and their corresponding classification levels in the comparison group D11 vs. D10.
