# Supplementary material for: Metabolomic Profiling Reveals the Effects of Cu-Ag Nanoparticles on Tomato Bacterial Wilt
Source: Metabolites. 2025 Aug 13;15(8):548. doi: 10.3390/metabo15080548 (PMC12388520; doi:10.3390/metabo15080548)
Supplement: Supplementary file 1 [file metabolites-15-00548-s001.zip › metabolites-3763775-supplementary.pdf]

Supplementary Materials

Metabolomic Profiling Reveals the Effects of Cu-Ag nanoparticles on Tomato Bacterial Wilt

Weimin Ning [1, 2, 3], Mei Yang [1], Tianhao Lei [1], Lei Jiang [1], Chan Liu [1], Fei Zhao [1], Pan Shu [1] *, Yong Liu [2,3,*]

[1] Agricultural Science College, Xichang University, Xichang 615000, China
[2] Longping Branch, Biology College, Hunan University, Changsha 410125, China
[3] Key Laboratory of Pest Management of Horticultural Crop of Hunan Province, Hunan Academy of Agricultural Science, Changsha 410125, China
* Correspondence: sp199546cau@163.com (P Shu); liuyong@hunaas.cn (Y Liu)

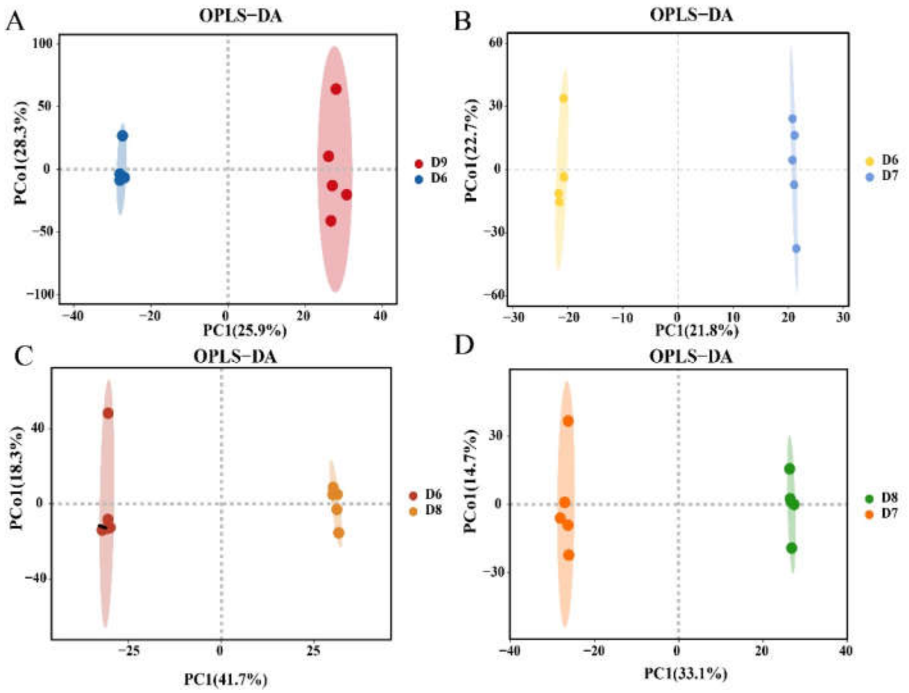

Figure S1. Score plot of orthogonal partial least squares discriminant analysis (OPLS-DA) of tomato root metabolites. D9 vs D6 (A). D7 vs D6 (B). D8 vs D6 (C). D7 vs D8 (D).

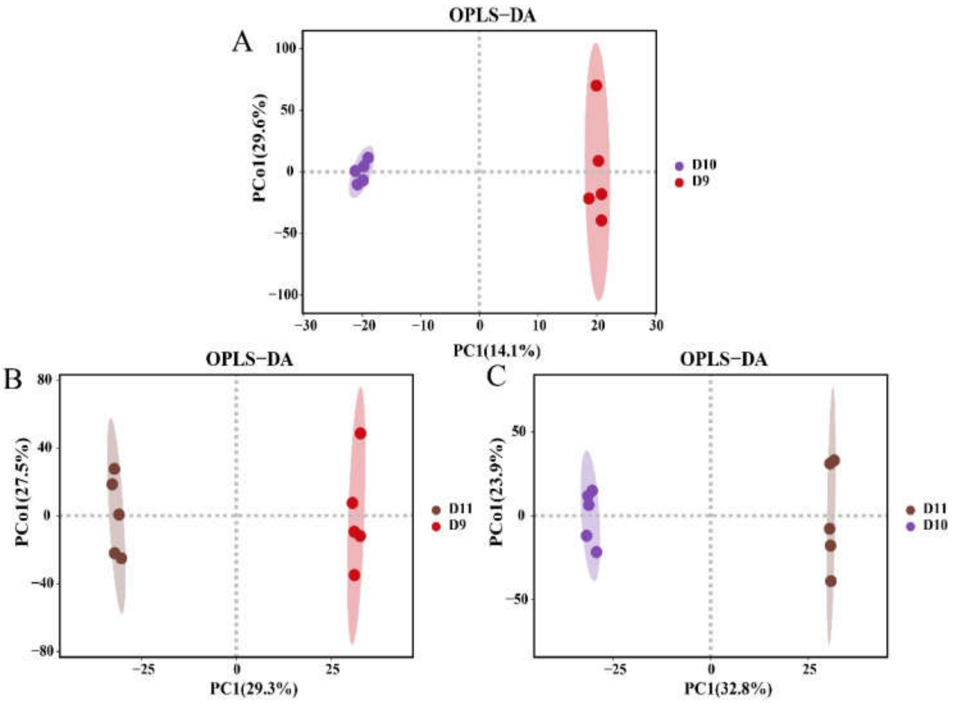

Figure S2. Score plot of orthogonal partial least squares discriminant analysis (OPLS-DA) of tomato root metabolites. D10 vs D9 (A). D11 vs D9 (B). D11 vs D10.

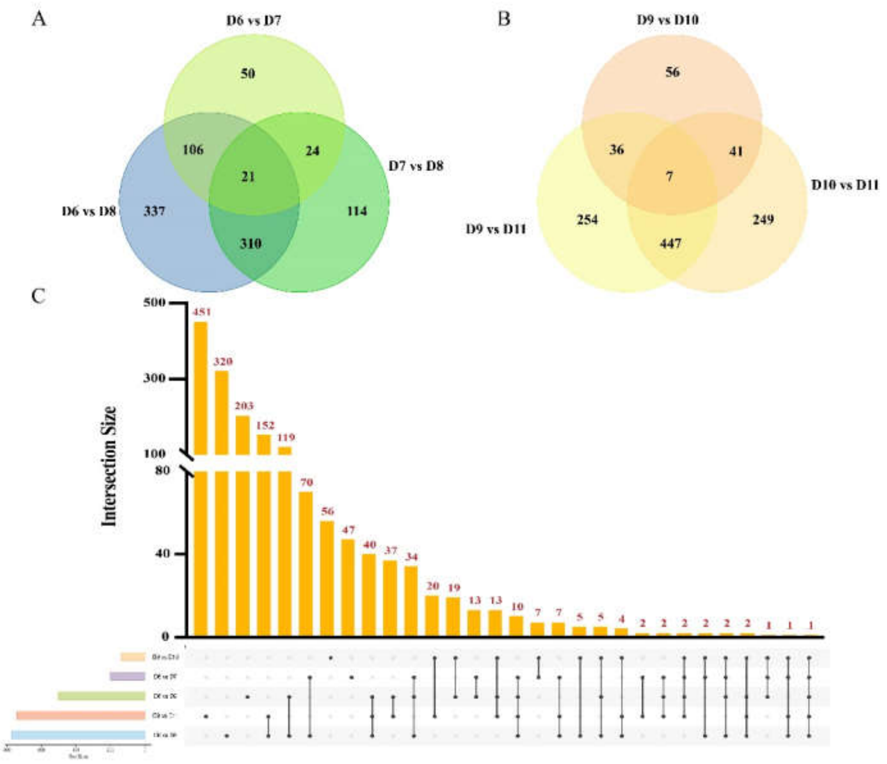

Figure S3. Venn diagram displaying unique and shared metabolites (DAMs) in diseased tomato roots (A) and healthy tomato roots (B). Upset diagram of DAMs in infected tomato roots and healthy roots (C).

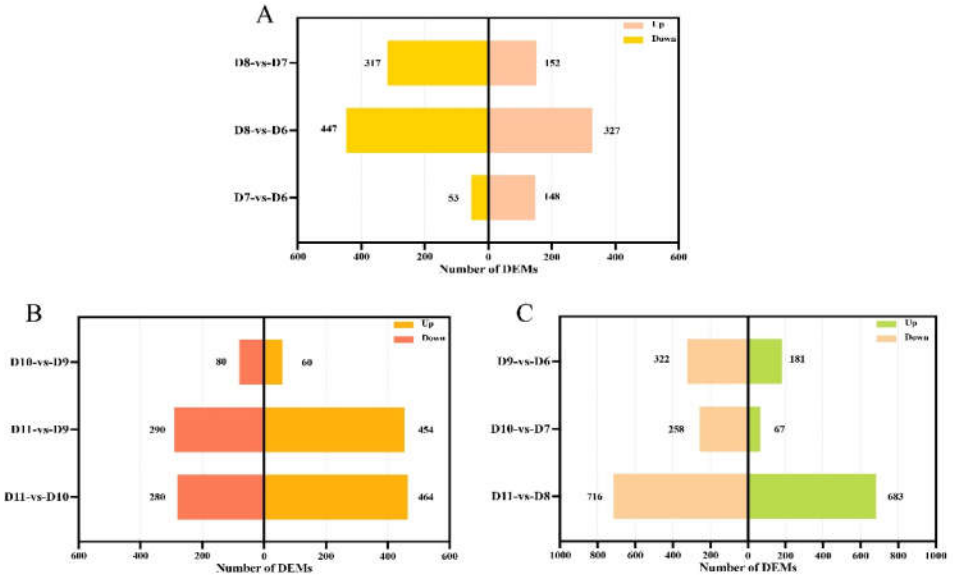

Figure S4. Analysis of differentially accumulated metabolites (DAMs) displaying up-regulation and down-regulation in sick tomato roots (A), healthy tomato roots (B), and the comparative groups of healthy and infected roots (C).

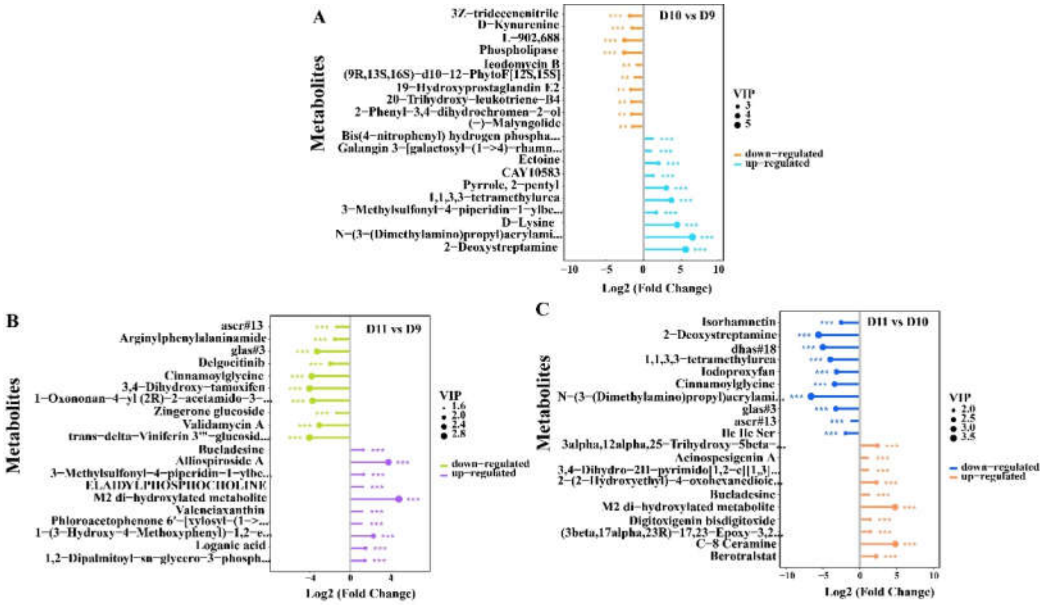

Figure S5. Analysis of the up-regulated and down-regulated metabolites in tomato roots using LollipopMap. D9 vs D10 (A). D9 vs D11 (B). D10 vs D11 (C).

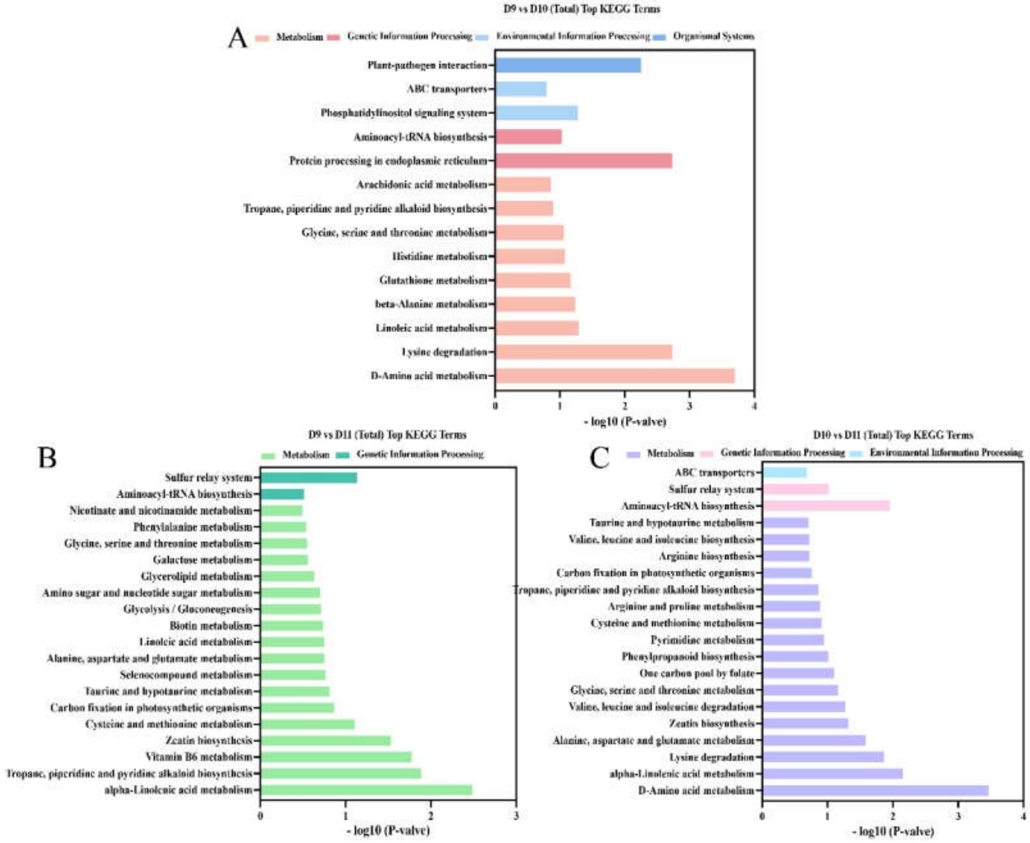

Figure S6. KEGG enrichment analysis of pathways in healthy tomato roots. The X-axis represents the -log10 p value of each pathway, and the Y-axis denotes the names of the various pathways. D9 vs D10 (A). D9 vs D11 (B). D10 vs D11 (C).

Table S1 Metabolites along with their respective classification levels in diseased tomato

Metabolites	Class
Daumone-2	Organooxygen compounds
Umbelliferone sulfate	Coumarins and derivatives
17-Aminogeldanamycin	Macrolactams
Quinceoxepine	Organooxygen compounds
Dihydronepetalactone	Prenol lipids
Roxatidine acetate	Piperidines
alpha-15,16-DiHODE	Fatty Acyls
Epicillin	Lactams
Chavicol	Phenols
Gibberellin A14	Prenol lipids
12,17	Fatty Acyls
Roxindole	Indoles and derivatives
R1-Barrigenol	Prenol lipids
17-hydroxy-linolenic acid	Fatty Acyls
(-)-Nopol	Prenol lipids
Rhubafuran	Benzene and substituted derivatives
Rhazidigenine Nb-oxide	Indoles and derivatives
Uvaretin	Diarylheptanoids

Table S1 Cont.

Metabolites	Class
5S-Hp-18R-HEPE	Fatty Acyls
(-)-11-Hydroxy-9,15,16-trioxooctadecanoic acid	Fatty Acyls
N-pentadecanoyl-L-Homoserine lactone	Carboxylic acids and derivatives
(-)-8,16-Dihydroxy-19-serrulatanoic acid	Prenol lipids
Plasmodiophorol A	Hydroxy acids and derivatives
Eremopetasitenin C1	Prenol lipids
Gallicynoic acid D	Fatty Acyls
FMLP	Carboxylic acids and derivatives
2-Deoxystreptamine	Organooxygen compounds
D-Lysine	Carboxylic acids and derivatives
N-(3-(Dimethylamino) propyl) acrylamide	Carboxylic acids and derivatives
Sarmentosin	Fatty Acyls
N-(4-(7-Diethylamino 4-methylcoumarin-3-yl) phenyl) iodoacetamide	Isoflavonoids
Acetamide, N-[4-[[(aminothioxomethyl) hydrazono] methyl] phenyl]-	Benzene and substituted derivatives
Apiferol	Flavonoids
(S)-2-Methyl-1-butanol O-beta-D-Glucopyranoside	Fatty Acyls
Isopentyl beta-D-glucoside	Fatty Acyls
2-Acetamido-2,6-dideoxygalactos	Organooxygen compounds

Table S1. Cont.

Metabolites	Class
3-Isopropylmalic acid	Fatty Acyls
4-oxo 2-Nonenal-d3	Organic oxygen compounds
gamma-9,10-DiHODE	Fatty Acyls
PGJ3	Fatty Acyls
9S-HOTrE	Fatty Acyls
Chembl4513510	Organoheterocyclic compounds
(2S,3R,4R,5S,6S)-2-(Hydroxymethyl)-6-phenylmethoxyoxane-3,4,5-triol1	Organooxygen compounds
1,1,3,3-tetramethylurea	Organic acids and derivatives
Pyrrole,2-pentyl	Pyrroles

Table S2 Metabolites along with their respective classification levels in healthy tomato

**Metabolites**	**Class**
Sodium decylbenzenesulfonate	Benzene and substituted derivatives
Berotralstat	Benzene and substituted derivatives
Geniposidic acid	Prenol lipids
Taccalonolide A	Steroids and steroid derivatives
Methyl 4,6-di-O-galloyl-beta-D-glucopyranoside	Tannins
Fluorometholone 17-acetate	Steroids and steroid derivatives
Valenciaxanthin	Prenol lipids
Dulciol B	Benzopyrans
Hecogenin	Prenol lipids
M2 di-hydroxylated metabolite	Indoles and derivatives
5'-Hydroxycarvedilol	Indoles and derivatives
Cinncassiol D2 glucoside	Prenol lipids
Bucladesine	Purine nucleotides
Tomatine	Steroids and steroid derivatives
Norfuraneol	Dihydrofurans
D-1,5-Anhydrofructose	Oxanes
6-O-(3R,4-dihydroxy-2-methylene-butanoyl)-beta-D-glucopyranose	Saccharolipids
Hovenidulcioside B1	Prenol lipids

Table S2. Cont.

Metabolites	Class
24-keto-25dehydrocholesterol	Steroids and steroid derivatives
Jurubine	Steroids and steroid derivatives
PC(6:2(3E,5E)/14:2(11E,13E))PE(0:0/22:6(4Z,7Z,10Z,13Z,16Z,19Z))	Glycerophospholipids
Apiferol	Flavonoids
1,2-Dipalmitoyl-sn-glycero-3-phospho-(1'D-myo-inositol-3'-phosphate)	Carboxylic acids and derivatives
6-O-(Glcb)-(25R)-5alpha-spirostan-3beta,6alpha-diol	Prenol lipids
Ile Ile Ser	Hydroxy acids and derivatives
oscr#9	Prenol lipids
Nafagrel	Organooxygen compounds
3-Methylsulfonyl-4-piperidin-1-ylbenzoyl guanidine	Piperidines
Pyrrole,2-pentyl	Pyrroles
N-(3-(Dimethylamino) propyl) acrylamide	Carboxylic acids and derivatives
D-Lysine	Carboxylic acids and derivatives
2-Deoxystreptamine	Organooxygen compounds
1,1,3,3-tetramethylurea	Organic carbonic acids and derivatives

Table S3 Regulation of metabolites and their corresponding classification levels in the comparison group D9 vs D6

**Classification_level2**	**Term**	**Up/down**
Lipid metabolism	alpha-Linolenic acid metabolism	down
Lipid metabolism	Glycerolipid metabolism	up
Lipid metabolism	Ether lipid metabolism	up
Metabolism of cofactors and vitamins	Thiamine metabolism	up
Metabolism of cofactors and vitamins	Pantothenate and CoA biosynthesis	down
Metabolism of cofactors and vitamins	Nicotinate and nicotinamide metabolism	up
Carbohydrate metabolism	Pentose phosphate pathway	up
Carbohydrate metabolism	Glyoxylate and dicarboxylate metabolism	down
Carbohydrate metabolism	Glycolysis / Gluconeogenesis	up
Amino acid metabolism	Histidine metabolism	down
Amino acid metabolism	Alanine, aspartate and glutamate metabolism	down
Metabolism of other amino acids	D-Amino acid metabolism	down
Metabolism of other amino acids	beta-Alanine metabolism	down
Energy metabolism	Carbon fixation in photosynthetic organisms	up
Energy metabolism	Oxidative phosphorylation	up
Nucleotide metabolism	Pyrimidine metabolism	down
Signal transduction	Phosphatidylinositol signaling system	down
Biosynthesis of other secondary metabolites	Tropane, piperidine and pyridine alkaloid biosynthesis	down
Environmental adaptation	Plant-pathogen interaction	down
Folding, sorting and degradation	Protein processing in endoplasmic reticulum	down

Table S4 Regulation of metabolites and their corresponding classification levels in the comparison group D7 vs D6

Classification_level2	Term	Up/down
Biosynthesis of other secondary metabolites	Biosynthesis of various alkaloids	down
Biosynthesis of other secondary metabolites	Flavonoid biosynthesis	up
Biosynthesis of other secondary metabolites	Tropane, piperidine and pyridine alkaloid biosynthesis	up
Biosynthesis of other secondary metabolites	Flavone and flavonol biosynthesis	down
Carbohydrate metabolism	Glyoxylate and dicarboxylate metabolism	up
Carbohydrate metabolism	Amino sugar and nucleotide sugar metabolism	up
Carbohydrate metabolism	Fructose and mannose metabolism	up
Carbohydrate metabolism	Pyruvate metabolism	up
Carbohydrate metabolism	Citrate cycle (TCA cycle)	up
Nucleotide metabolism	Pyrimidine metabolism	down
Nucleotide metabolism	Purine metabolism	up
Lipid metabolism	Glycerophospholipid metabolism	up
Lipid metabolism	Glycerolipid metabolism	up
Amino acid metabolism	Glycine, serine and threonine metabolism	up
Amino acid metabolism	Arginine biosynthesis	up
Amino acid metabolism	Valine, leucine and isoleucine biosynthesis	up
Amino acid metabolism	Lysine degradation	up
Amino acid metabolism	Alanine, aspartate and glutamate metabolism	up
Metabolism of other amino acids	D-Amino acid metabolism	up
Metabolism of other amino acids	Glutathione metabolism	down

Table S5 Regulation of metabolites and their corresponding classification levels in the comparison group D8 vs D6

**Classification_level2**	**Term**	**Up/down**
Carbohydrate metabolism	Amino sugar and nucleotide sugar metabolism	down
Carbohydrate metabolism	Starch and sucrose metabolism	up
Carbohydrate metabolism	Pyruvate metabolism	up
Carbohydrate metabolism	Pentose and glucuronate interconversions	down
Carbohydrate metabolism	Fructose and mannose metabolism	up
Metabolism of other amino acids	Glutathione metabolism	up
Metabolism of other amino acids	Taurine and hypotaurine metabolism	up
Metabolism of cofactors and vitamins	Thiamine metabolism	up
Metabolism of cofactors and vitamins	Pantothenate and CoA biosynthesis	up
Metabolism of cofactors and vitamins	Nicotinate and nicotinamide metabolism	down
Energy metabolism	Sulfur metabolism	up
Energy metabolism	Oxidative phosphorylation	up
Amino acid metabolism	Cysteine and methionine metabolism	up
Amino acid metabolism	Valine, leucine and isoleucine biosynthesis	up
Lipid metabolism	Ether lipid metabolism	up
Lipid metabolism	alpha-Linolenic acid metabolism	down
Glycan biosynthesis and metabolism	N-Glycan biosynthesis	down
Folding, sorting and degradation	Sulfur relay system	up
Nucleotide metabolism	Pyrimidine metabolism	down
Biosynthesis of other secondary metabolites	Tropane, piperidine and pyridine alkaloid biosynthesis	down

Table S6 Regulation of metabolites and their corresponding classification levels in the comparison group D8 vs D7

**Classification_level2**	**Term**	**Up/down**
Amino acid metabolism	Cysteine and methionine metabolism	up
Amino acid metabolism	Lysine degradation	down
Amino acid metabolism	Alanine, aspartate and glutamate metabolism	down
Amino acid metabolism	Glycine, serine and threonine metabolism	down
Amino acid metabolism	Arginine biosynthesis	down
Metabolism of other amino acids	Cyanoamino acid metabolism	up
Metabolism of other amino acids	Taurine and hypotaurine metabolism	up
Metabolism of other amino acids	D-Amino acid metabolism	down
Metabolism of other amino acids	Glutathione metabolism	down
Lipid metabolism	Ether lipid metabolism	up
Lipid metabolism	alpha-Linolenic acid metabolism	down
Biosynthesis of other secondary metabolites	Flavone and flavonol biosynthesis	up
Biosynthesis of other secondary metabolites	Tropane, piperidine and pyridine alkaloid biosynthesis	down
Metabolism of terpenoids and polyketides	Terpenoid backbone biosynthesis	up
Metabolism of terpenoids and polyketides	Zeatin biosynthesis	down
Metabolism of cofactors and vitamins	Thiamine metabolism	up
Metabolism of cofactors and vitamins	Pantothenate and CoA biosynthesis	up
Folding, sorting and degradation	Sulfur relay system	up
Energy metabolism	Sulfur metabolism	up
Glycan biosynthesis and metabolism	N-Glycan biosynthesis	down

Table S7 Regulation of metabolites and their corresponding classification levels in the comparison group D10 vs D9

Classification_level2	Term	Up/down
Metabolism of other amino acids	Glutathione metabolism	up
Metabolism of other amino acids	beta-Alanine metabolism	up
Metabolism of other amino acids	D-Amino acid metabolism	up
Lipid metabolism	Arachidonic acid metabolism	down
Lipid metabolism	Linoleic acid metabolism	down
Amino acid metabolism	Glycine, serine and threonine metabolism	up
Amino acid metabolism	Histidine metabolism	up
Amino acid metabolism	Lysine degradation	up
Biosynthesis of other secondary metabolites	Tropane, piperidine and pyridine alkaloid biosynthesis	up
Translation	Aminoacyl-tRNA biosynthesis	up
Signal transduction	Phosphatidylinositol signaling system	up
Environmental adaptation	Plant-pathogen interaction	up
Folding, sorting and degradation	Protein processing in endoplasmic reticulum	up
Membrane transport	ABC transporters	up

Table S8 Regulation of metabolites and their corresponding classification levels in the comparison group D11 vs D9

Classification_level2	Term	**Up/down**
Carbohydrate metabolism	Amino sugar and nucleotide sugar metabolism	down
Carbohydrate metabolism	Galactose metabolism	up
Carbohydrate metabolism	Glycolysis / Gluconeogenesis	up
Metabolism of cofactors and vitamins	Nicotinate and nicotinamide metabolism	up
Metabolism of cofactors and vitamins	Biotin metabolism	down
Metabolism of cofactors and vitamins	Vitamin B6 metabolism	down
Metabolism of other amino acids	Selenocompound metabolism	down
Metabolism of other amino acids	Taurine and hypotaurine metabolism	down
Amino acid metabolism	Phenylalanine metabolism	down
Amino acid metabolism	Glycine, serine and threonine metabolism	down
Amino acid metabolism	Cysteine and methionine metabolism	down
Amino acid metabolism	Alanine, aspartate and glutamate metabolism	down
Lipid metabolism	Glycerolipid metabolism	up
Lipid metabolism	Linoleic acid metabolism	down
Lipid metabolism	alpha-Linolenic acidmetabolis	up
Folding, sorting and degradation	Sulfur relay system	down
Metabolism of terpenoids and polyketides	Zeatin biosynthesis	down
Biosynthesis of other secondary metabolites	Tropane, piperidine and pyridine alkaloid biosynthesis	up

Table S8. Cont.

Classification_level2	Term	Up/down
Translation	Aminoacyl-tRNA biosynthesis	down
Energy metabolism	Carbon fixation in photosynthetic organisms	down

Table S9 Regulation of metabolites and their corresponding classification levels in the comparison group D11 vs D10

**Classification_level2**	**Term**	**Up/down**
Metabolism of otheraminoacids	Taurine and hypotaurine metabolism	down
Metabolism ofother aminoacids	D-Amino acid metabolism	down
Biosynthesis of other secondary metabolites	Tropane, piperidine and pyridine alkaloid biosynthesis	down
Biosynthesis of other secondary metabolites	Phenylpropanoid biosynthesis	down
Amino acid metabolism	Arginine and proline metabolism	down
Amino acid metabolism	Cysteine and methionine metabolism	down
Amino acid metabolism	Glycine, serine and threonine metabolism	down
Amino acid metabolism	Arginine biosynthesis	down
Amino acid metabolism	Valine, leucine and isoleucine biosynthesis	down
Amino acid metabolism	Valine, leucine and isoleucine degradation	down
Amino acid metabolism	Lysine degradation	down
Amino acid metabolism	Alanine, aspartate and glutamate metabolism	down
Energy metabolism	Carbon fixation in photosynthetic organisms	down
Folding, sorting and degradation	Sulfur relay system	down
Metabolism of cofactors and vitamins	One carbon pool by folate	down
Membrane transport	ABC transporters	down
Metabolism of terpenoids and polyketides	Zeatin biosynthesis	down
Translation	Aminoacyl-tRNA biosynthesis	down

Table S9. Cont.

**Classification_level2**	**Term**	**Up/down**
Nucleotide metabolism	Pyrimidine metabolism	up
Lipid metabolism	alpha-Linolenic acid metabolism	up
